# Application of Protection Motivation Theory to Quantify the Impact of Pandemic Fear on Anticipated Postpandemic Transit Usage

**DOI:** 10.1177/03611981211065439

**Published:** 2022-02-21

**Authors:** Sk. Md. Mashrur, Kaili Wang, Patrick Loa, Sanjana Hossain, Khandker Nurul Habib

**Affiliations:** 1 Department of Civil & Mineral Engineering, University of Toronto, Toronto, ON, Canada; 2 Percy Edward Hart Professor in Civil & Mineral Engineering, University of Toronto, Toronto, ON, Canada

**Keywords:** planning and development, public transportation, transit

## Abstract

The COVID-19 pandemic had an unprecedented impact on transit usage, primarily owing to the fear of infection. Social distancing measures, moreover, could alter habitual travel behavior, for example, using transit for commuting. This study explored the relationships among pandemic fear, the adoption of protective measures, changes in travel behavior, and anticipated transit usage in the post-COVID era, through the lens of protection motivation theory. Data containing multidimensional attitudinal responses about transit usage at several pandemic stages were utilized for the investigation. They were collected through a web-based survey in the Greater Toronto Area, Canada. Two structural equation models were estimated to examine the factors influencing anticipated postpandemic transit usage behavior. The results revealed that people taking relatively higher protective measures were comfortable taking a cautious approach such as complying with transit safety policies (TSP) and getting vaccinated to make transit trips. However, the intention to use transit on vaccine availability was found to be lower than in the case of TSP implementation. Conversely, those who were uncomfortable taking transit with caution and who were inclined to avoid travel and rely on e-shopping were most unlikely to return to transit in the future. A similar finding was observed for females, those with vehicle access, and middle-income individuals. However, frequent transit users during the pre-COVID period were more likely to continue to use transit after the pandemic. The study’s findings also indicated that some travelers might be avoiding transit specifically because of the pandemic, implying they are likely to return in the future.

The novel Coronavirus disease 2019 (COVID-19) has generated an unprecedented level of fear of infection among trip makers, with public transit undoubtedly suffering the most. Several studies reported that transit ridership dropped by as much as 90% because of the pandemic (*1, 2*). Although part of this decline in ridership can be attributed to the reduction in commuting trips associated with work-from-home policies, part of it is also related to the perceived risk and fear of getting infected while using transit. For example, several studies have revealed people’s risk perception of public transit to be greater than that of private modes during the pandemic, potentially affecting their transit usage behavior (*3, 4*). Therefore, transportation planners and transit service providers have been continually challenged to develop effective policies to recover the lost ridership as the pandemic situation improves.

The perceived fear and the resulting motivation to adopt protective measures could potentially alter transit usage behavior in the postpandemic era (*5*). Protective measures undertaken in response to the pandemic are likely to be maintained for an extended period as a precaution after the pandemic. Consequently, some individuals may exhibit inertia, that is, a delayed return to transit as long as these measures are in place. As a result, public transit ridership might be adversely affected from these precautions. Using simulation results, a study conducted in New York found that such behavioral inertia would result in postpandemic transit ridership being 27% lower than prepandemic levels, even with no restrictions on transit capacity (*6*). At the same time, it was depicted that vehicular trips may increase by as much as 42%. Failure to recover transit demand might therefore bring additional challenges to the postpandemic transportation system, such as congestion. These findings indicated that protective measures include avoiding travel, using e-shopping as a substitute for traveling, and shifting to alternate modes from transit (*7*). Therefore, it is critical to understand individuals’ behaviors during the pandemic and then use the insights to inform effective policies to ensure the postpandemic sustainability of public transit systems.

This study investigated the behavioral consequences of pandemic fear on anticipated postpandemic transit usage in the Greater Toronto Area (GTA), Ontario, Canada. Specifically, this study utilized the theoretical framework of protection motivation theory to explore the relationships between pandemic fear, the adoption of protective measures, travel behavior changes, and anticipated transit usage in the post-COVID era. The study considered the travel behavior changes of the inclination to avoid travel, to use transit cautiously, and to rely on e-shopping during the pandemic. The relationships between these factors were investigated using structural equation models (SEM). Two models were estimated considering attitudes toward transit usage under two travel contexts: a situation where transit safety policies (TSPs) have been implemented in response to the pandemic and another where the COVID-19 vaccine is readily available. The study results will shed light on the effects of implementing health and safety policies in transit systems and the availability of vaccines to recover lost transit ridership in the post-COVID era.

The remainder of the paper is organized as follows: The next section presents the theoretical background and the hypotheses tested in this study. Then the context of the research, including the data collection process and descriptive statistics are summarized. Later, the paper presents the empirical framework adopted for the investigation, followed by a discussion of the results and the potential policy implications of the findings. Finally, the main conclusions of the study and directions for future research are highlighted.

## Theoretical Background and Hypotheses

COVID-19 and its impact on passenger travel demand have triggered an avalanche of research within the transportation planning community. Most of the studies focused on the alteration of travel behaviors caused by the pandemic (*8–15*). Their findings all confirmed that the pandemic had changed the behaviors of travelers worldwide. As transit demand plunged, some studies also specifically studied transit demand. It was observed that passengers perceiving a higher risk of influenza infection in public transit were more likely to avoid transit trips (*16, 17*). Likewise, such concerns posed by the pandemic were found to incline individuals to use private vehicles (*18*). Nevertheless, within the literature, several researchers investigated travelers’ willingness to use transit after implementing several transit-related health and safety measures (*19–22*). They found that most travelers held positive attitudes toward safety measures, such as social distancing, mask mandates, crowd management, cleanliness, and intended to take transit. Another notable study surveyed eight Chinese cities where transit services were suspended during the peak of the pandemic in China (*23*). The study revealed the mechanisms of how transit riders perceived their satisfaction of transit service during the recovery period of the pandemic. Using structural equation modeling, they found that riders’ anxiety negatively affected their perceived safety and eventually led to dissatisfaction with transit. However, to the authors’ knowledge, only a small number of studies have explored the potential for travelers’ psychological factors (i.e., fear of pandemic and consequent motivation to adopt protective measures) and behavioral intentions (i.e., inclination to avoid travel, online shopping) to alter their postpandemic transit usage behavior. Thus, this study aimed to address this research gap.

### Fear of the Pandemic

In response to the threat posed by COVID-19, governments worldwide have implemented mobility restrictions, temporarily closed businesses and schools, and instituted social distancing guidelines (*24, 25*). Although these policies were implemented to protect public health, they also have the potential to affect people’s perceptions of the risks associated with the pandemic. Previous studies have shown that latent attitudinal factors and perceptions can affect mode choice (*26, 27*), meaning that the pandemic and related policies could affect the utilization of public transit. Additionally, based on protection motivation theory, individuals may turn to adaptive coping mechanisms (which aim to protect against threats) in response to a public health threat (*5*). Given the potential for perceptions of risk and fear to affect the use of public transit during the pandemic, further investigation is needed to determine whether these factors will affect post-COVID public transit usage.

Greater perceptions of risk associated with a threat (including public health threats) typically increase the propensity to adopt protective measures (*28–31*). As outlined in protection motivation theory, an individual’s perception of a threat is influenced by two factors: the perceived severity of the threat and their perceived vulnerability to it (*32*). Previous studies have shown that fear can influence the relationship between an individual’s attitude toward a threat and their adoption of protective behaviors, often leading to an increase in their motivation to adopt said behaviors (*5, 33, 34*). For example, the impact of so-called pandemic travel fear on postpandemic tourism was investigated by Zheng et al. (*5*). Their results suggested that the perceived consequences of infection, the health threat to tourists, the risk of being infected, and the possibility of exposure to infected individuals all contributed to greater levels of travel fear. In this study, the term *pandemic fear* is defined as a reflection of the individual’s concern toward different aspects of the pandemic and their beliefs about the risks associated with leaving one’s home during the pandemic. Based on previous work on the topic, pandemic fear may lead individuals to adopt protective measures, leading to the following hypothesis:

H1: Greater pandemic fear will result in greater motivation to adopt protective measures.

### Behavioral Intentions

Behavioral intentions are considered a key indicator of one’s future behaviors (*35*). Several marketing researchers have regarded behavioral intentions as a measurement of customer loyalty in purchasing and promoting specific services (*36*). However, many have argued that intentions to use a service might not lead to action, and frequent usage does not always reveal intentions (*37*). Many transit users might be using transit repeatedly because they have limited alternative modes to choose from. A study investigating changes in travel behavior in GTA during the COVID-19 pandemic reported that among frequent transit users prepandemic, those with access to private vehicles were more likely to have avoided transit during the pandemic than the group without private vehicle access (*38*). Therefore, it is suggested that user loyalty be regarded as a combination of behavioral attitudes such as intention to use the service again and willingness to use it despite the availability of a new alternative (*39*).

Adopting protective measures in response to the pandemic might lead to behavioral changes, including the initiation of new behaviors or the cessation or alteration of existing behaviors (*32*). Previous studies on tourism revealed that individuals might avoid traveling as a direct protective measure, or travel cautiously when faced with travel fear triggered by a health crisis. Furthermore, studies suggest that uptake of protective measures can be a good indicator in explaining such attitudes (*5*). In addition, several measures, including the replacement of out-of-home activities with online activities, adopting the use of face coverings when using public transit, and refraining from using public transit during peak hours, have been discussed in the literature (*15*).

With this in mind, this study incorporated two behavioral measures as indicators of travel avoidance: the inclination to avoid short- and long-distance travel and the inclination to rely on online shopping. Online shopping might replace some transit trips, since people can make purchases online without traveling to the stores, limiting the risk of infection. Conversely, the inclination to use transit considered two contexts: implementation of transit health and safety policies, and vaccine availability. These behavioral attitudes were subsequently used to examine postpandemic transit usage behavior. Therefore, the study examined the following hypotheses:

H2a: The greater the motivation to adopt protective measures, the greater the inclination to avoid travel.
H2b: The greater the motivation to adopt protective measures, the greater the inclination to use transit cautiously.
H2c: The greater the motivation to adopt protective measures, the greater the inclination to rely on online shopping.

**Figure 1.**
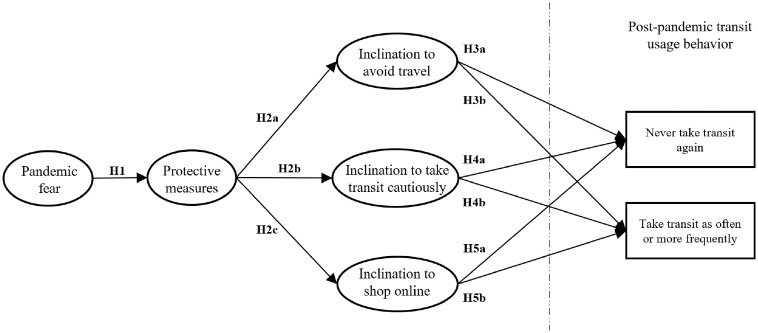
Conceptual model to investigate the impact of COVID-19 fear on postpandemic transit usage behavior.

These protective travel behaviors could potentially affect postpandemic transit usage behavior, such as strictly avoiding transit or using it as often or more frequently than as in the prepandemic period. Thus, the propensity for travel avoidance during the pandemic might lead to less frequent transit usage. Conversely, an optimistic attitude toward using transit while following transit safety protocols and in the case of being vaccinated might be a positive sign for recovering lost transit ridership post-COVID. Thus, this study aimed to investigate to what extent the tendency to use transit postpandemic is influenced by (i) a tendency to avoid travel (H3); (ii) an inclination to use transit (H4); and (iii) a reliance on e-shopping (H5). The conceptual model is illustrated in Figure 1.

H3a: The greater the inclination to avoid travel, the greater the tendency to never use transit again.
H3b: The greater the inclination to avoid travel, the lower the tendency to use transit as often or more frequently than in the prepandemic era.
H4a: The greater the inclination to use transit cautiously, the lower the tendency never to use transit.
H4b: The greater the inclination to use transit cautiously, the greater the tendency to use transit as often or more frequently than in the prepandemic era.
H5a: The greater the inclination to rely on online shopping, the greater the tendency to never use transit.
H5b: The greater the inclination to rely on online shopping, the lower the tendency to use transit as often or more frequently than in the prepandemic era.

## The Survey

### Survey Design and Data Collection

**Figure 2.**
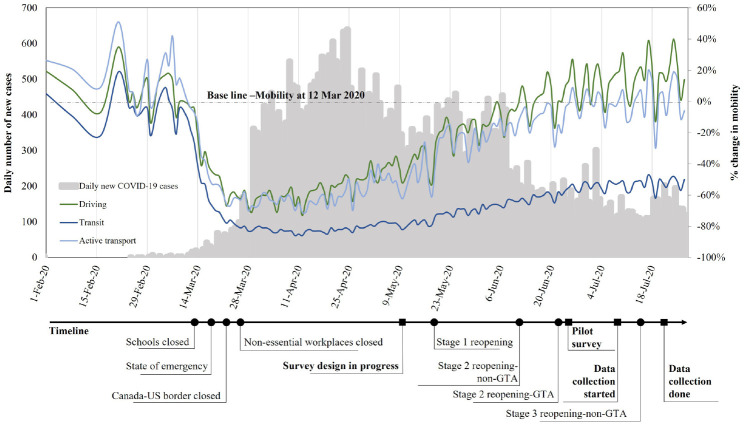
Daily number of new cases in Ontario and the Apple Mobility trends in Toronto, Ontario (*40*).

This study used data from the project Stated Preference Experiment on Travel mode and especially Transit choice behavior (SPETT) that aimed to capture the COVID impact on transit usage in GTA, Canada during the summer of 2020 (*38*). The project collected the data through a market research company, which randomly sent an invitation to the members of their consumer panel residing in GTA. However, a residential quota was imposed to ensure consistent distribution among the sample and the population in GTA. Later, the respondents were remunerated with nonmonetary incentives by the market research company for the time required to complete the survey. The final dataset used for empirical investigation in this study contained 933 records after data cleaning. The survey collected information on respondents’ personal and household socioeconomic characteristics, transit usage before and during the pandemic, and various transit-related attitudinal variables. Hereafter, the terms *before, during*, and *postpandemic/future* refer to the following periods, respectively: before the declaration of the state of emergency, at the time of data collection, and the period when COVID-19 will not be considered a threat. Figure 2 illustrates the change in mobility trends during the first pandemic wave in Ontario, with some critical dates shown (*40, 41*). At the time of data collection, the first wave of the pandemic was in a declining phase with decreasing numbers of daily COVID cases (i.e., 14-day average cases ranged from 162 to 178). No vaccines were officially approved for administration in the region at that point. Figure 2 clearly indicates that transit usage plummeted significantly just after the restrictions were enacted. Moreover, the recovery rate for lost transit demand was very low compared with private vehicles and active transport.

The distribution of key socioeconomic statistics was compared against a reference dataset: the 2016 Transportation Tomorrow Survey (TTS). The TTS is a regional household travel survey conducted every 5 years that has covered GTA since 1986 (*42*). The 2016 TTS was expanded with respect to the 2016 Canadian census; thus, the socioeconomic characteristics in the 2016 TTS represent the exact characteristics of the population in GTA. Most key statistics of the 2020 SPETT survey matched reasonably well with the characteristics of the population in the study area. Table 1 presents the socioeconomic variables from the samples collected through the survey. The distribution of driver’s license holders, household size, household vehicles, and household incomes matched the population’s characteristics and were within 5% of the reference dataset. However, the dataset slightly overrepresented females and residents from the Toronto, York, and Peel regions, which have wider transit coverage than other regions. The dataset also contained a higher percentage of transit pass holders before the pandemic than the general population. Given the study’s objective, such an overrepresentation of frequent transit users and respondents residing in regions with well-served transit coverage may have been beneficial.

**Table 1.** Summary Statistics of the 2020 SPETT Survey

Variables	2020 SPETT (%)	2016 TTS (%)
Age		
18–29	21	20
30–39	25	18
40–49	20	19
50–59	17	20
>60	17	23
Gender		
Male	41	48
Female	59	52
Driver’s license holders	86	82
Transit pass holders before the pandemic	37	24
Employment status		
Full-time	59	54
Part-time	15	11
Not employed	26	35
Student status		
Full-time	10	7
Part-time	6	3
Not a student	84	90
Household size		
1	15	24
2	28	28
3	23	17
4	22	18
5+	12	12
Household vehicle		
0	11	16
1	45	40
2	35	33
3+	9	11
Household location by regions		
Toronto	40	48
Durham	8	10
York	23	15
Peel	24	19
Halton	5	8
Household income		
<$15,000	2	5
$15,000–39,999	11	14
$40,000–59,999	15	14
$60,000–99,999	28	21
$100,000–124,999	13	10
>$124,999	20	18

*Note*: SPETT = Stated Preference Experiment on Travel mode and especially Transit choice behavior; TTS = Transportation Tomorrow Survey.

### Descriptive Statistics

#### Postpandemic Transit Usage Behavior

**Figure 3.**
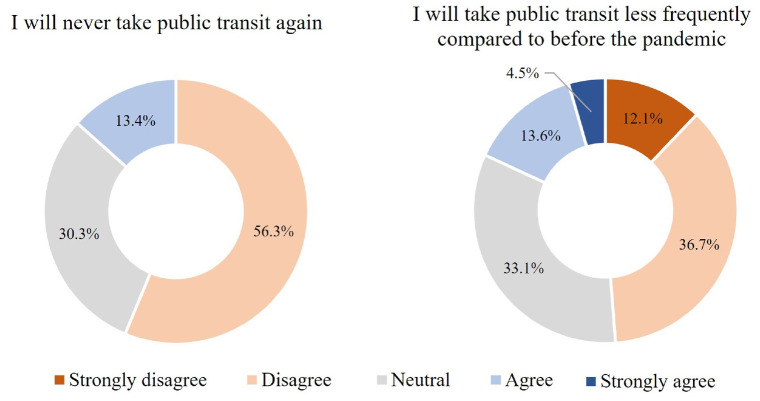
Respondents’ attitudes toward postpandemic public transit usage.

Respondents were asked to report their attitudes toward postpandemic transit usage. Figure 3 presents a summary of their responses. Some 56.3% of the respondents disagreed that they would never use transit again, as opposed to 13.4% who firmly agreed to this statement. In addition, 48.8% of the respondents disagreed that they would be using transit less frequently postpandemic. These observations paint an optimistic picture about transit ridership recovery when the pandemic is over. They further indicated that the transit ridership drop experienced during the pandemic might just be temporary.

#### Pandemic Concerns and Protective Measures

**Figure 4.**
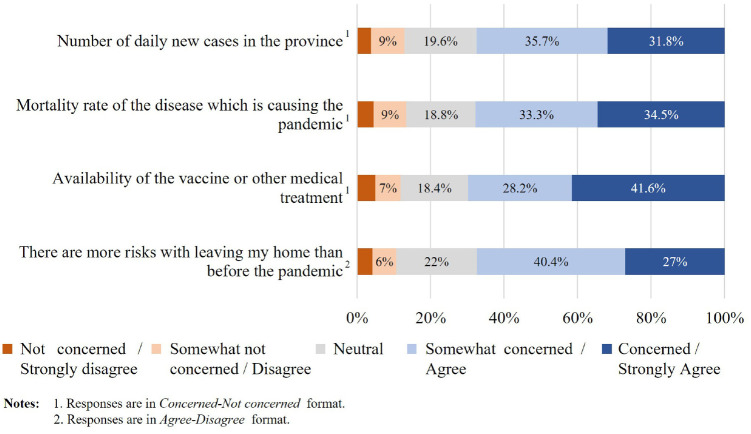
Distribution of responses to attitudinal questions about pandemic fear.

**Figure 5.**
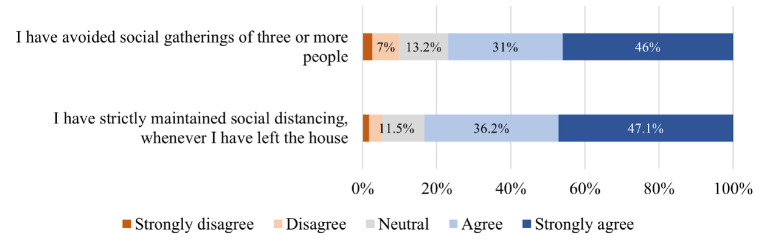
Distribution of responses to protective measures questions.

The results showed that most respondents were concerned about the pandemic (see Figure 4). For example, 67.4% of respondents believed that there were more risks associated with leaving their homes during the pandemic. Furthermore, a similar proportion of respondents was concerned about the number of daily new cases reported in Ontario, the mortality rate, and the availability of vaccines or any medical treatments to fight COVID-19. The extent of the respondents’ practice of protective measures during the pandemic was also captured (Figure 5): 83.3% reported that they strictly practiced social distancing when they left home; 77% stated that they avoided gatherings of three or more people to protect themselves against COVID-19.

#### Travel Avoidance and E-Shopping Reliance

**Figure 6.**
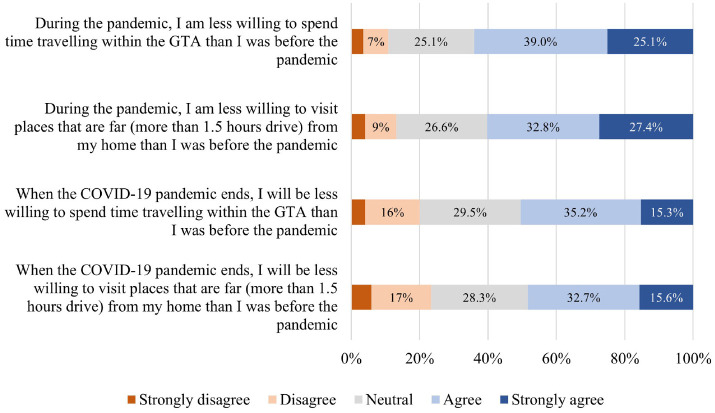
Distribution of responses to attitudinal questions on travel avoidance.

Governments worldwide have restricted mobility to curb the spread of COVID-19 during the pandemic (*43*). Moreover, the study showed that most respondents were disinterested in traveling during the pandemic (see Figure 6). Over 60% were less willing to make both short- and long-distance trips in that period. Moreover, enforcement of mobility restrictions during the pandemic might also have long-term impacts on travel behavior. Notably, 48.3% reported that they might be less willing to travel even after the pandemic.

**Figure 7.**
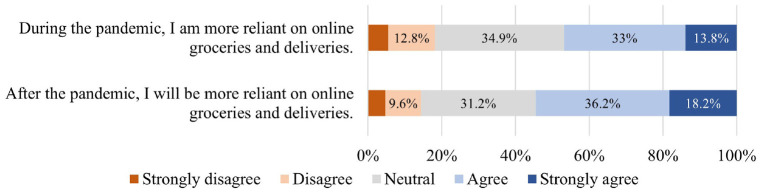
Distribution of responses to e-shopping-reliance attitudes.

People developed and increased their reliance on e-shopping during the pandemic (*44*). Statistics Canada reported that e-shopping sales doubled in May 2020, 1 month after the spread of COVID-19 had been seen in Canada (*44*). Figure 7 represents respondents’ attitudes on their e-shopping reliance during and after the pandemic: 46.8% reported being more reliant on e-shopping during the pandemic, and 54.4% believed they will continue to rely on this even after the pandemic.

#### Cautious Transit Usage

**Figure 8.**
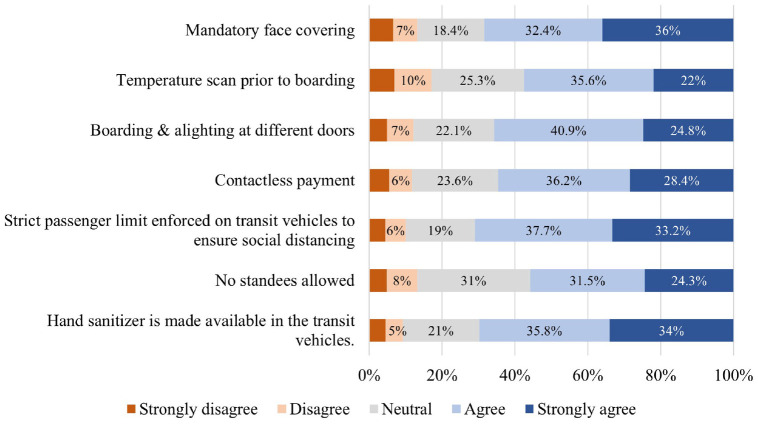
Distribution of responses to cautious transit usage questions considering transit safety policies.

Respondents also reported their attitudes toward various TSPs, put in place to protect riders during the pandemic (see Figure 8). Overall, policies were welcomed by more than 60% of the respondents, except for *No standees allowed* (55.8% agreed) and *Temperature scan before boarding* (57.6% agreed). Nonetheless, more than 50% of the respondents reported they would feel safe using transit again, given that the listed policies would be in place.

**Figure 9.**
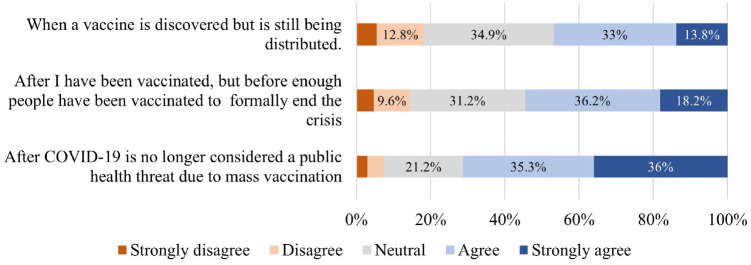
Distribution of responses to transit usage questions considering vaccine availability.

Mass vaccination is regarded as one way to end the pandemic (*43*). Respondents were asked to indicate whether they would be willing to use transit during the several stages of the pandemic specifically in relation to vaccination. The results indicated that respondents’ willingness to return to public transit increased with the progression of mass vaccination (see Figure 9). Once mass vaccination is attained, 71.3% of respondents firmly agreed that they would return to public transit.

## Methodology

### Empirical Model

This study utilized SEM to examine the hypotheses presented in Theoretical Background and Hypotheses. SEM is a multivariate statistical technique that is widely used to develop empirical models to understand the diverse interrelations among variables of interest (*45*). SEM has two components. The first component, the measurement model, incorporates confirmatory factor analysis. The analysis explains the covariations of observed indicators defining the latent construct, and measures the correlations among the constructs. Once the measurement model is specified, the structural equations define the interrelations between the latent constructs. Further detailing of the model can be found in the literature (*46*). In this study, the maximum likelihood estimation method was used for estimating the model using the lavaan package in the statistical computing software R (*47*).

The study also adopted five standard indices to assess model fit: the ratio of the chi-square statistic to the degrees of freedom ( $\chi^{2}$ /df), the root mean square error of approximation (RMSEA), the Tucker–Lewis index (TLI), the comparative fit index (CFI), and the standardized root mean square residual (SRMSR). The threshold limits of these measures for a good model fit are $\chi^{2}$ /df < 5, RMSEA < 0.08, SRMSR < 0.08, TFI > 0.90, and CFI > 0.95. Additionally, a minimum sample size of 200 is considered satisfactory for SEM analyses (*46, 48*).

### Construct Measures

The latent constructs were informed by protection motivation theory to ensure their subjective validity. To quantify pandemic fear, respondents were asked to rate their level of concern about several aspects of the pandemic. These included the number of daily new cases in the province, the disease’s mortality rate, and the availability of a vaccine or other medical intervention to deal with COVID-19. Additionally, an attitudinal question was asked to elicit the anxiousness associated with leaving the house during the pandemic. These indicators were validated to reference and scale the *Pandemic fear* construct (*49–51*). The response option for the first three indicators was in a concerned–not concerned format, whereas the anxiousness indicator was in an agree–disagree format. The indicators for the two-item *Protective measures* latent construct were selected based on previous protection behavior studies, and responses were recorded in an agree–disagree format (*5, 52*).

The constructs, *Inclination to avoid travel, E-shopping inclination*, and *Inclination to take transit cautiously*, were adopted to examine changes in users’ loyalty to transit services post-COVID. To measure the first two constructs, respondents were provided with two cases: during the pandemic (the data collection period), and the time frame when COVID-19 is no longer considered a public health threat (*37, 53, 54*). For the *Inclination to avoid travel* construct, respondents were asked to indicate their unwillingness to spend time on short- and long-distance travel. Respondents were also asked to report their reliance on online orders and deliveries for the *E-shopping inclination* construct. Finally, the *Inclination to take transit cautiously* construct was measured considering respondents’ transit usage attitudes for two travel environments: implementation of feasible TSPs, and vaccine availability. This construct refers to the extent to which the respondents felt safe to take transit under the given environments. The questions for the corresponding indicators used an agree–disagree response format. All the observed indicators discussed in this section were measured using a five-point Likert scale (“1”: not concerned/strongly disagree, “5”: concerned/strongly agree); the distribution of responses is illustrated in Figures 4 to 9.

### Measurement Model

Common method bias was examined using Harman’s one-factor test and the nonresponse bias test (*55*). Using the former test, factor analysis was carried out without rotation (*56*). The analysis indicated that the singular factor explained only 25.4% of the total variance, far below the 50% threshold (*57*). For the latter test, the sample was subdivided into two halves by the collected response date. The difference in the responses between the first and second half of the respondents was insignificant at a 90% confidence level, signifying the absence of nonresponse bias in the dataset (*58*). Both results indicated that common method bias was not evident.

Construct reliability and validity tests were also conducted. The results are shown in Table 2. The factor loading of the constructs’ indicators was observed to be within 0.405 to 0.950, above the cutoff value of 0.40 (*46*). All but one construct (*Protective measures*) had Cronbach’s alpha (α) and composite reliability values exceeding the threshold of 0.70 (*59*). However, there are studies suggesting that α values within 0.60 and 0.70 are acceptable for exploratory studies (*46, 60*).

**Table 2.** Summary Statistics and Factor Loadings of the Observed Indicators on the Latent Constructs

Latent constructs	Observed indicator		Mean	SD	Factor loading
Pandemic fear (α = 0.822)	pF_1	Number of daily new cases in the province	3.83	1.09	**0.865**
	pF_2	The mortality rate of the disease that is causing the pandemic	3.84	1.13	**0.888**
	pF_3	Availability of the vaccine or other medical treatment	3.95	1.15	**0.798**
	pF_4	There are more risks with leaving my home than before the pandemic	3.80	1.04	**0.405**
Protective measures (α = 0.682)	pM_1	I have avoided social gatherings of three or more people	4.07	1.12	**0.724**
	pM_2	I have strictly maintained social distancing whenever I have left the house	4.19	1.00	**0.719**
Inclination to avoid travel (α = 0.817)	tA_1	During the pandemic, I am less willing to spend time traveling within GTA than I was before the pandemic	3.75	1.02	**0.594**
	tA_2	When the COVID-19 pandemic ends, I will be less willing to spend time traveling within GTA than I was before the pandemic	3.42	1.06	**0.860**
	tA_3	During the pandemic, I am less willing to visit places that are far (more than 1.5-h drive) from my home than I was before the pandemic	3.70	1.09	**0.584**
	tA_4	When the COVID-19 pandemic ends, I will be less willing to visit places that are far (more than 1.5-h drive) from my home than I was before the pandemic	3.35	1.12	**0.833**
Inclination to take transit cautiously (transit safety policies) (α = 0.931)	tsp_1	Mandatory face covering	3.85	1.18	**0.824**
	tsp_2	Temperature scan before boarding	3.55	1.14	**0.757**
	tsp_3	Boarding and alighting at different doors to lessen the interaction between riders	3.73	1.06	**0.861**
	tsp_4	Contactless payment	3.76	1.10	**0.835**
	tsp_5	Strict passenger limit enforced on transit vehicles to ensure social distancing	3.90	1.07	**0.855**
	tsp_6	No standees allowed	3.62	1.09	**0.737**
	tsp_7	Hand sanitizer is made available in transit vehicles	3.90	1.07	**0.820**
Inclination to take transit cautiously (vaccine availability) (α = 0.717)	vac_1	After I have been vaccinated, but before enough people have been vaccinated to formally end the crisis	3.54	1.04	**0.655**
	vac_2	After COVID-19 is no longer considered a public health threat because of to mass vaccination	3.97	1.01	**0.854**
E-shopping inclination (α = 0.834)	eS_1	During the pandemic, I am more reliant on online groceries and deliveries	2.98	1.27	**0.754**
	eS_2	After the pandemic, I will be more reliant on online groceries and deliveries	2.90	1.23	**0.950**

*Note*: SD = standard deviation. *p* < 0.001 are presented in boldface.

Additionally, all the constructs were subjected to two validity tests: convergent validity and discriminant validity (presented in Table 3). The average variance extracted (AVE) for the constructs exceeded the AVE threshold of 0.50. For the discriminant validity, the square root of the AVE of each construct was calculated and compared with the correlation among the constructs. All the square roots were observed to be higher than the intercorrelation among the constructs. The results confirmed the validity of the accounted constructs in this study (*46*).

**Table 3.** Validity Test Results of the Latent Constructs

Latent constructs	Composite reliability	AVE	Discriminant validity					
Pandemic fear	0.840	0.598	**0.775**	0.532	0.261	0.279	0.241	0.078
Protective measures	0.685	0.521	0.532	**0.721**	0.368	0.335	0.191	0.118
Inclination to avoid travel	0.815	0.536	0.261	0.368	**0.735**	0.191	0.003	0.458
Inclination to take transit cautiously (transit safety policies)	0.932	0.661	0.279	0.335	0.191	**0.812**	0.357	0.067
Inclination to take transit cautiously (vaccine availability)	0.732	0.574	0.241	0.191	0.003	0.357	**0.755**	–0.075
E-shopping inclination	0.846	0.731	0.078	0.118	0.458	0.067	-0.075	**0.854**

*Note*: AVE = average variance extracted.

AVE square roots are underlined and boldface.

## Results and Discussion

**Figure 10.**
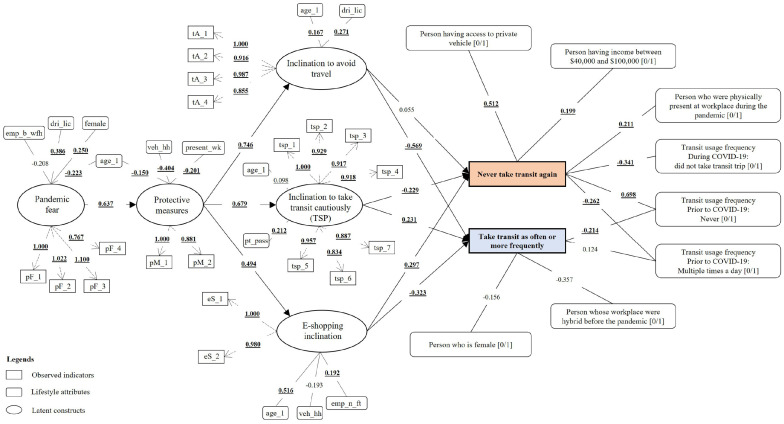
Structural equation model (SEM) estimates for Model 1 (*p* < 0.05 are in boldface and underlined).

**Figure 11.**
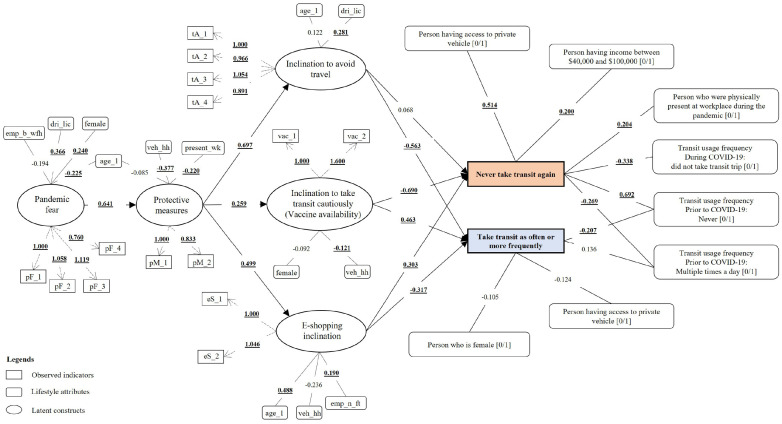
Structural equation model (SEM) estimates for Model 2 (*p* < 0.05 are in boldface and underlined).

The study estimated two SEMs for investigating postpandemic transit usage behavior considering two categories of the *Inclination to take transit cautiously* construct, as discussed in the “Construct Measures” section. Model 1 (M1) accounted for potential TSPs as a driving force to encourage passengers to take transit, whereas Model 2 (M2) considered the impact of vaccination in recovering transit demand. Two indicators defined the postpandemic transit usage in the models. One referred to strict transit avoidance, and the other denoted a certain degree of future transit usage. The first indicator considered the respondent’s level of intent to *Never take transit again* in the future as an indication of a negative travel attitude. The response options for the indicator followed a three-point agree–disagree response format (1 = disagree to 3 = agree) and accounted for the participant’s attitudes toward reduced transit usage in the future compared with the pre-COVID-19 period. This was measured on a five-point Likert scale, using the agree–disagree format. However, the corresponding responses to the question were reverse coded in the model to denote positive travel behavior, termed *Taking transit as often or more frequently*. In addition to examining the hypothesized model shown in Figure 1, the paper also investigated the correlation between socioeconomic attributes, latent constructs (shown in Table 4), and the postpandemic transit usage indicators. The model results are summarized in Table 5, and the path diagrams with full results are illustrated in Figures 10 and 11.

**Table 4.** Socioeconomic Attributes Influencing the Latent Constructs

			Model 1: Transit safety policy		Model 2: Vaccine availability	
Latent constructs	Socioeconomic attributes		β	*t*-stat	β	*t*-stat
Pandemic fear	age_1	Age: 18 to 34	**−0.223**	−3.17	**−0.225**	−3.28
	female	Gender: Female	**0.250**	3.34	**0.240**	3.46
	dri_lic	Holding a driver’s license	**0.386**	3.30	**0.366**	3.38
	emp_b_wfh	Before COVID-19, workplace was home	−0.208	−1.66	−0.194	−1.64
Protective measures	age_1	Age: 18 to 34	**−0.150**	−2.28	−0.085	−1.32
	veh_hh	Vehicle(s) per household member	**−0.404**	−3.73	**−0.377**	−3.40
	present_wk	Physically present at the workplace during the pandemic	**−0.201**	−2.79	**−0.220**	−3.12
Inclination to avoid travel	age_1	Age: 18 to 34	**0.167**	2.25	0.122	1.77
	dri_lic	Holding a driver’s license	**0.271**	2.72	**0.281**	2.82
Inclination to take transit cautiously	age_1	Age: 18 to 34	0.098	1.23	—	—
	pt_pass	Having a transit pass during the pandemic	**0.212**	2.43	—	—
	female	Gender: Female	—	—	−0.092	−1.81
	veh_hh	Vehicle(s) per household member	—	—	−**0.121**	−2.06
E-shopping inclination	age_1	Age: 18 to 34	**0.516**	5.46	**0.488**	5.35
	veh_hh	Vehicle(s) per household member	−0.193	−1.40	−0.236	−1.83
	emp_n_ft	During the pandemic, working full-time	**0.192**	2.17	**0.190**	2.22

*Note: p* < 0.05 are presented in boldface.

**Table 5.** Factors Affecting Postpandemic Transit Usage Attitudes

Hypothesis				Model 1: Transit safety policy				Model 2: Vaccine availability			
				β		*t*-stat		β		*t-*stat	
H1	Pandemic fear	→	Protective measures	**0.637**		10.37		**0.641**		11.26	
H2a	Protective measures	→	Inclination to avoid travel	**0.746**		9.41		**0.697**		10.02	
H2b	Protective measures	→	Inclination to take transit cautiously	**0.679**		8.41		**0.259**		4.48	
H2c	Protective measures	→	E-shopping inclination	**0.494**		6.17		**0.499**		6.49	
				Never take transit again		Take transit as often or more frequently		Never take transit again		Take transit as often or more frequently	
				β	t-stat	β	t-stat	β	t-stat	β	t-stat
H3a	Inclination to avoid travel	→	Never take transit again	0.055	0.99	—	—	0.068	1.18	—	—
H4a	Inclination to take transit cautiously	→	Never take transit again	−**0.229**	−5.19	—	—	−**0.690**	−6.91	—	—
H5a	E-shopping inclination	→	Never take transit again	**0.297**	7.29	—	—	**0.303**	7.53	—	—
H3b	Inclination to avoid travel	→	Take transit as often or more frequently	—	—	**-0.569**	−12.41	—	—	−**0.563**	−11.81
H4b	Inclination to take transit cautiously	→	Take transit as often or more frequently	—	—	**0.231**	7.37	—	—	**0.463**	6.50
H5b	E-shopping inclination	→	Take transit as often or more frequently	—	—	**-0.323**	−9.43	—	—	−**0.317**	−9.57
Transit usage frequency				β	*t*-stat	β	*t*-stat	β	*t*-stat	β	*t*-stat
frq_pt_b_0	Before COVID-19: Never			**0.698**	5.91	**-0.214**	−2.07	**0.692**	5.94	−**0.207**	−2.03
frq_pt_b_1	Before COVID-19: Multiple times a day			**−0.262**	−1.99	0.124	1.06	**-0.269**	−2.05	0.136	1.17
frq_pt_n_0	During COVID-19: Did not take transit			**-0.341**	−3.04	—	—	**-0.338**	−3.13	—	—
Socioeconomic attributes				β	*t*-stat	β	*t*-stat	β	*t*-stat	β	*t*-stat
female	Gender: Female			—	—	−0.156	−1.90	—	—	−0.105	−1.34
pv_acc	Having access to private vehicle			**0.512**	2.87	—	—	**0.51**	2.88	−0.124	−0.82
inc_mid	Income: $40,000–100,000			**0.199**	2.23	—	—	**0.20**	2.25	—	—
Employment attributes				β	*t*-stat	β	*t*-stat	β	*t*-stat	β	*t*-stat
emp_b_hyb	Before COVID-19, the workplace was hybrid			—	—	−0.357	−1.94	—	—	—	—
present_wk	Physically present at the workplace during the pandemic			**0.211**	2.21	—	—	**0.204**	2.14	—	—
Threshold parameters				β	*t*-stat	β	*t*-stat	β	*t*-stat	β	*t*-stat
1→2				**0.810**	4.06	**-1.755**	−9.34	**0.795**	4.06	**-1.723**	−9.43
2→3				**1.797**	8.82	**-0.594**	−3.36	**1.782**	8.86	**-0.565**	−3.29
3→4				—	—	**0.366**	2.06	—	—	**0.392**	2.28
4→5				—	—	**1.202**	6.31	—	—	**1.226**	6.57
Fit indices											
$\chi^{2}$				965.843				902.441			
Degrees of freedom (df)				450				263			
$\chi^{2}$ /df				2.146				3.431			
Comparative fit index (CFI)				0.950				0.879			
Tucker–Lewis index (TLI)				0.977				0.945			
Root mean square error of approximation (RMSEA)				0.039				0.057			
Standardized root mean square residual (SRMSR)				0.071				0.090			

*Note*: hybrid = being allowed to work at both their usual workplace and at home.

*Never take transit again* has a three-scale-, and *Take transit more frequently* has a 5-scale response option.

*p* < 0.05 are presented in boldface.

Both the models’ fit indices were within the acceptable limit as discussed in the “Empirical Model” section, except for the CFI and SRMSR of M2. However, considering the study’s exploratory nature and taking all the goodness-of-fit measures together, it could be implied that the models fit the data for conducting the said investigation (*61, 62*). Whereas most of the attributes in the models were significant at a 95% confidence level, some insignificant parameters were retained owing to their behavioral insights into future transit usage.

### Socioeconomic Factors and Latent Constructs

The relationship between socioeconomic factors (representing lifestyle characteristics) and the latent constructs was considered in both the models (see Table 4). It was observed that younger adults displayed less pandemic fear and had a lower tendency to take protective measures. Moreover, they had a higher propensity for shopping online. The findings aligned with social psychology studies that concluded that young adults perceive themselves to be at a lower risk of COVID infection and are therefore less likely to embrace protective measures (*63*). However, young adults were found to acknowledge the severity of the disease for the community. The e-shopping inclination for the said group may be explained by their higher degree of tech-savviness (*64*). Female respondents were observed to have higher pandemic fear. These outcomes were consistent with earlier studies (*65*).

In relation to mobility tool ownership, respondents with a transit pass during the pandemic were seen to take transit in cases in which TSPs were implemented. One explanation for this could be that these individuals had a positive attitude toward the effectiveness of the safety policies against infection, or they might have had limited modal alternatives, which led them to hold a transit pass during the pandemic. Conversely, higher vehicle ownership was associated with a lower propensity to take protective measures, take transit, and shop online. The findings are intuitive, as vehicle ownership gives the individual the freedom to avoid shared spaces and the flexibility to schedule activities at his/her convenience (i.e., going to a grocery shop when deemed to be less crowded). Moreover, such attitudes toward e-shopping for this group have been validated by prior studies (*66*). However, those holding a driver’s license perceived higher pandemic fear and displayed a greater inclination to avoid travel.

The models further depicted that those working from home before the pandemic percieved a lower degree of pandemic fear. Those who had to be present at the workplace during the pandemic were found to have a lower tendency to take protective measures. However, a higher tendency to shop online was observed in those working full-time either from home, in the workplace, or under hybrid arrangements (defined as being allowed to combine working at both their usual workplace and at home) during the pandemic. This might indicate a full-time worker’s intention to avoid additional shopping trips, to limit their exposure amidst the pandemic.

### Causal Effects

The study tested the 10 aforementioned hypothesized causal relationships. The model results shown in Table 5 were consistent with the protection motivation theory, stating that higher pandemic fear persuaded the respondents to take more protective measures. This endorsed H1 and echoed the earlier research findings that had adopted the theory to examine COVID-19’s impact on tourism (5). In addition, it was observed that individuals’ protective measures significantly affected their travel avoidance inertia, the inclination to take transit cautiously, and e-shopping inclination, which validated H2a, H2b, and H2c. The models further disclosed that individuals with a higher propensity for travel avoidance and online shopping during the pandemic were more inclined to never use transit again, substantiating hypotheses H3a and H5a. Conversely, those who felt comfortable making transit trips under the safety policies implemented during the pandemic had an optimistic attitude toward making similar or more transit trips than before the pandemic in the future. A similar attitude was observed for those with a higher propensity to use transit on vaccine availability. Moreover, these groups were also less likely to completely avoid transit in the future. These findings supported hypotheses H4a and H4b. Both the models found all the hypotheses except for H3a to be statistically significant (*p* < 0.001).

From M1, it was revealed that the higher the level of protective measures taken by the respondents, the greater their propensity to take transit trips when the safety policies were implemented. This finding also aligned with previous studies that concluded that riders are willing to take transit and to pay more on the implementation of various measures, such as providing more frequent services to avoid crowded vehicles (*19–22*). The findings also suggested that health-conscious trip makers trusted the safety policies’ effectiveness in minimizing infection during transit. This might be because of the proven effectiveness of the health and safety policies implemented in curbing the spread of COVID-19 (*67*). Moreover, the assurance provided by transit agencies implementing policies such as crowd control and smart card payment can build confidence in passengers (*68*). In doing so, transit demand can be induced, which might partially offset the cost of implementing such measures. Likewise, M2 results indicated that individuals who had a higher propensity to embrace protective measures were also likely to take transit in the case of vaccine availability. However, this tendency was lower compared with TSP adoption. This may be because of the respondents’ skepticism about the effectiveness of vaccines in containing the virus or concerns about the potential adverse side effects of the vaccine (*69*). At the time of data collection, COVID-19 vaccines had yet to be developed. However, both the models concluded that individuals inclining to use transit cautiously were more likely to return to their prepandemic transit usage behavior post-COVID.

### Lifestyle and Future Transit Usage

Aside from the discussed factors, the models also investigated the influence of respondents’ transit usage patterns before and during the pandemic on their anticipated postpandemic transit usage attitude. It was found that individuals who had never used transit for their daily trips before the pandemic would continue to not do so when the pandemic ends. This finding is intuitive, considering the additional vigilance on hygiene concerns posed by the pandemic. Similar findings were observed in relation to concerns about getting infected while taking transit trips (*16, 17*). However, those who took transit trips frequently before the pandemic were more likely to use transit regularly or more frequently postpandemic. Surprisingly, the models indicated that those who did not make transit trips during the pandemic might also return to transit after the pandemic. This finding signifies the prospect of an increase in transit demand when COVID-19 is no longer a public health threat. Previous studies on the impacts of COVID-19 also argued that the reduction in transit trips could mainly be attributed to restricted mobility, working from home, and other social distancing strategies (*38*).

Female respondents were found to be more likely to take transit in the future but were more likely to take transit at a lower frequency than their prepandemic usage. However, individuals who had private vehicle access and were from middle-income households ($40,000–$100,000) were found to be more likely to avoid transit completely in the future. These findings echoed prior research findings (*22*). The results also concluded that workers with hybrid workplace arrangements before the pandemic would prefer to use transit less frequently when the pandemic ends. However, respondents who were workplace-based during the pandemic (e.g., essential workers) were more inclined to never take transit in the future. Further analysis of the data showed that more than 90% of workers who belonged to these two groups had access to private vehicles. This made the correlations relatable to other model outcomes about the postpandemic transit usage attitudes of the vehicle access group. These findings raise concerns over increased autodependency during the postpandemic period (*6, 18*)

## Policy Implications

The model results suggested that adopting TSPs in response to the pandemic somewhat offset the adverse effects of pandemic fear on transit demand. Such approaches appear to have encouraged passengers to take transit trips during the postpandemic period. Thus, the utmost importance should be given to policies that mitigate infection risk while taking transit trips. At the same time, transit agencies should opt for policies that do not substantially limit vehicle capacity; otherwise, transit agencies will incur operational costs from the additional runs required to maintain favorable conditions (e.g., limiting increases in waiting times for trip makers). One possible solution could be to strictly mandate personal protective measures such face coverings while taking transit. In addition, as riders are now more vigilant about hygiene, policies ensuring such safety measures should be emphasized. Frequent cleaning of vehicles, the availability of hand sanitizer, and contactless payment systems are among the many prominent hygiene safety protocols that could be implemented. Additionally, notifying riders about the vehicles’ and station’s cleanliness by any visible means possible, could help to gain trust in the transit system’s enhanced hygiene protocols. For example, a variable message sign showing the last time the vehicle was disinfected or indicating its frequency per day might gain trust among riders.

However, amidst the pandemic, a situation might arise when transit agencies will be required to adopt policies to assure social distancing in the transit vehicles, which will indeed limit vehicle capacity. One solution might be to provide real-time transit vehicle crowd information in such a scenario, which is now becoming popular in developed cities like Toronto (*70*). In that way, riders can schedule their activity in light of this information. However, from the operational side, some of the less used routes could be merged during the pandemic, allowing the unused vehicle runs to be reallocated to higher-demand routes. Moreover, transit service providers could decide to keep specific stops or whole routes, in light of pandemic-generated practices such as online shopping and working from home. One such decision might be whether to operate the routes connecting shopping centers as regularly as in the prepandemic period or to rework service plans considering the altered demand at specific stops. It was observed that online shopping has the prospect to reduce transit demand for shopping trips.

The models further depicted that regular transit users in the prepandemic era were willing to use transit as often or more frequently in the post-COVID context. This is indicative of the role of TSPs in helping to rebuild transit demand during the pandemic. The intention of regular prepandemic transit users to use transit as frequently in the post-COVID period would suggest that transit demand will rebound once COVID-19 is no longer a public health threat. Thus, transit agencies should strive to provide reliable, safe service in the future to not lose transit demand from those willing to return.

Attention should also be paid to accelerating vaccine administration, as it was found to positively affect postpandemic transit usage intentions, even though some are skeptic about the vaccine’s potential effectivity. Public health authorities should build confidence in city residents with respect to the effectiveness of the approved vaccines so that the community on mass rapidly gets vaccinated on its availability. The sooner residents are vaccinated, the sooner the daily new cases drop, along with mortality (*71*). In turn, this will aid ramping up transit ridership recovery.

## Conclusions and Future Work

This study investigated the impact of pandemic fear and the protective measures induced by the COVID-19 pandemic on anticipated transit usage behavior once the pandemic is over. The study utilized data collected from a web-based survey conducted in GTA. Two SEMs were developed considering TSPs and vaccine availability as driving forces to attract passengers to use transit again. In addition, the inclination to avoid travel and a reliance on e-shopping, which increased following pandemic-related social distancing measures, were also investigated to examine their effect on postpandemic transit usage.

The results showed that those taking greater protective measures displayed a higher propensity for travel avoidance and shopping online in the future. However, they also felt safer using transit cautiously when TSPs were adopted and vaccines were available to ensure protection against infection. However, individuals taking more protective measures were seen to have a reduced intention to use transit on vaccine availability than with TSP adoption.

The study further revealed that individuals with a higher propensity for travel avoidance and e-shopping during the pandemic were more inclined to avoid transit. However, attitudes toward taking transit trips in a cautious environment (i.e., following TSPs and being vaccinated) had a positive impact on the probability of using transit as often or more frequently once the pandemic is over. It was observed that frequent prepandemic transit users, along with those who did not take transit trips during the pandemic, were also optimistic about using transit in the future. However, those who had vehicle access, belonged to the medium income-group, and were workplace-based during the pandemic showed a higher propensity for never using transit post-COVID.

The relationship between socioeconomic attributes and latent constructs was also examined. Younger adults were seen to have lower fear perception and a reduced tendency to take protective measures. Additionally, they had a higher propensity for taking transit trips and shopping online. Conversely, female respondents were observed to have higher sensitivity toward pandemic fear and a lower propensity for taking transit. Respondents using mobility tools such as transit passes were more likely to take transit, whereas the opposite was observed for those with higher vehicle ownership. The latter group also had a negative attitude toward online shopping. However, those who were working full-time during the pandemic were found to be inclined to e-shopping.

Despite providing valuable insights, the study has some limitations. First of all, the survey was collected online during the recovery period of the first wave of the pandemic, when the severity of the subsequent pandemic waves was still unknown. Moreover, owing to the online data collection methodology, the survey respondents were more likely to be younger and tech-savvy than the study area’s general population. Such a discrepancy has the potential to affect model outcomes. Secondly, the study considered only the prospect of e-shopping (i.e., online groceries and deliveries) as a competing alternative to travel. Other alternatives such as on-demand services (e.g., Uber eats, Postmates) and telecommuting should also be assessed to have better insight into the competition. The second cycle of the survey will provide valuable insights in this regard. Lastly, the study only applied protection motivation theory to examine postpandemic transit usage behavior. Other theories such as coping, resilience theories, cultural differences, and media impact also need to be incorporated to better perceive individuals’ transit usage behavior (*5*). Furthermore, a comprehensive mode choice model incorporating telecommuting and online shopping needs to be developed that considers the several influential factors related to the pandemic. Thus, transit agencies will have a clearer picture of what to expect in a post-COVID context and will be able to make conclusive decisions in recovering the lost demand.
